# 6,6′-Dieth­oxy-2,2′-[propane-1,2-diyl­bis(nitrilo­methyl­idyne)]diphenol

**DOI:** 10.1107/S1600536809003328

**Published:** 2009-02-28

**Authors:** Zhen Jia

**Affiliations:** a Department of Chemistry, Dezhou University, Dezhou 253023, People’s Republic of China

## Abstract

In the title mol­ecule, C_21_H_26_N_2_O_4_, the dihedral angle between the two benzene rings is 88.4 (3)°. Two fairly strong intra­molecular O—H⋯N hydrogen bonds may, in part, influence the mol­ecular conformation.

## Related literature

For background information on the coordination ability of tetradentate Schiff-base ligands, see: Bermejo *et al.* (2007 ▶); Ni *et al.* (2005 ▶); Nayak *et al.*, 2006 ▶; Mohanta *et al.*, 2002 ▶; Saha *et al.* (2007 ▶); Wang *et al.* (2008 ▶); Yu *et al.* (2007 ▶). 
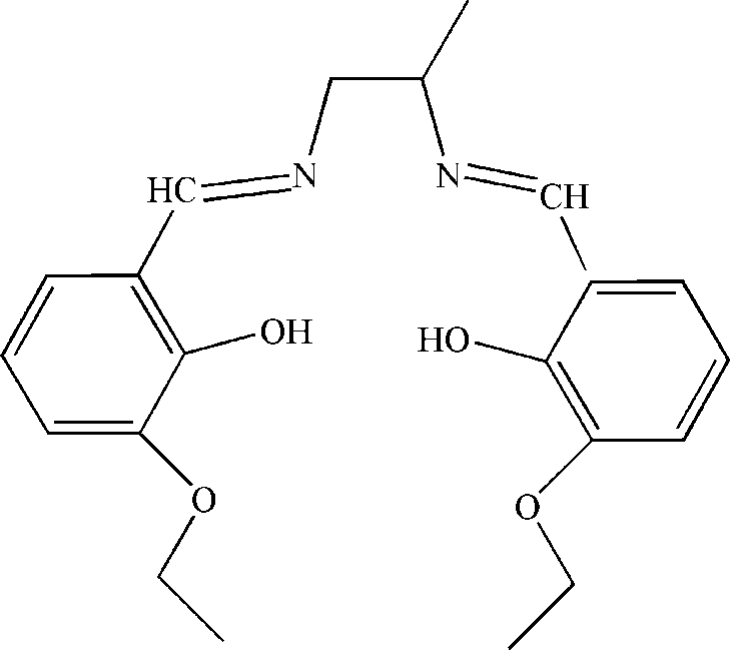


## Experimental

### 

#### Crystal data

C_21_H_26_N_2_O_4_
*M* *_r_* = 370.44
Triclinic, 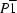

*a* = 9.140 (3) Å
*b* = 11.451 (4) Å
*c* = 13.013 (5) Å
α = 113.845 (5)°
β = 109.628 (6)°
γ = 108.812 (5)°
*V* = 993.5 (6) Å^3^
*Z* = 2
Mo *K*α radiation
μ = 0.09 mm^−1^
*T* = 273 (2) K
0.12 × 0.11 × 0.09 mm

#### Data collection

Bruker APEXII CCD area-detector diffractometer
Absorption correction: multi-scan (*SADABS*; Sheldrick, 1996 ▶) *T*_min_ = 0.990, *T*_max_ = 0.992
4894 measured reflections
3451 independent reflections
2325 reflections with *I* > 2σ(*I*)
*R*_int_ = 0.030

#### Refinement

*R*[*F* ^2^ > 2σ(*F* ^2^)] = 0.066
*wR*(*F* ^2^) = 0.193
*S* = 1.00
3451 reflections
249 parameters
H-atom parameters constrained
Δρ_max_ = 0.26 e Å^−3^
Δρ_min_ = −0.26 e Å^−3^

### 

Data collection: *APEX2* (Bruker, 2004 ▶); cell refinement: *SAINT-Plus* (Bruker, 2001 ▶); data reduction: *SAINT-Plus*; program(s) used to solve structure: *SHELXS97* (Sheldrick, 2008 ▶); program(s) used to refine structure: *SHELXL97* (Sheldrick, 2008 ▶); molecular graphics: *SHELXTL* (Sheldrick, 2008 ▶); software used to prepare material for publication: *SHELXTL*.

## Supplementary Material

Crystal structure: contains datablocks I, global. DOI: 10.1107/S1600536809003328/lh2762sup1.cif

Structure factors: contains datablocks I. DOI: 10.1107/S1600536809003328/lh2762Isup2.hkl

Additional supplementary materials:  crystallographic information; 3D view; checkCIF report

## Figures and Tables

**Table 1:** Hydrogen-bond geometry (Å, °)

*D*—H⋯*A*	*D*—H	H⋯*A*	*D*⋯*A*	*D*—H⋯*A*
O3—H3⋯N2	0.82	1.89	2.614 (3)	147
O1—H1⋯N1	0.82	1.85	2.576 (2)	146

**Fig. 1.**
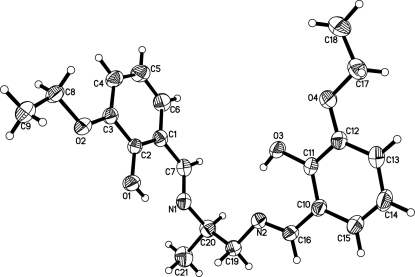
The molecular structure of the title compound with the atom-labelling scheme. Displacement ellipsoids are drawn at the 30% probability level.

## supplementary crystallographic information

### Comment

Among the tetradentate schiff-base ligands studied intensely during the past several decades, the planar, salen types and their complexes (with interesting magnetic properties) have been given much attention due to their excellent coordination ability, utilizing two N atoms and two O atoms (Ni *et al.*, 2005; Wang *et al.*, 2008; Yu *et al.*, 2007; Nayak *et al.*, 2006; Mohanta *et al.*, 2002; Saha *et al.*, 2007).

Herein, we report the synthesis and crystal structure of a new tetradentate Schiff base ligand, *N*,*N*'-bis(2-hydroxy-3-ethoxybenzylidene)- 1,2-diaminopropane. The molecular structure of the title compound is shown in Fig. 1. The molecule possesses an O_2_N_2_ donor set affording a potentially tetradentate ligand. The imide bond lengths 1.262 (3) for N1—C7 and 1.263 (3) Å for N2—C16 are slightly shorter than that of *N*,*N*'-bis(2-hydroxy-3-methoxybenzylidene)ethylenediamine (1.272 Å) (Bermejo, *et al.*, 2007). The C—N bond distance of 1.461 (3) Å is in agreement well with that found in the same compound. Two fairly strong intramolecular O···H—N hydrogen bonds may in part influence the molecular conformation.

### Experimental

The Schiff base ligand was synthesized by condensation 1,2-diaminopropane and 2-hydroxy-3-ethoxybenzaldehyde with molar ratio 1:2 in ethanol. The mixture formed was allowed to partially evaporate in air for sevral days to produce crystals suitable for X-ray diffraction with a yield about 65%.

### Refinement

H atoms were placed in calculated positions with C—H distances of 0.93, 0.96, 0.97 Å and O—H 0.82 Å, and were allowed for as riding atoms with *U*_iso_(H) = 1.5*U*_eq_(C) for methyl H atoms, *U*_iso_(H) = 1.2*U*_eq_(C) for all other C bound H atoms and *U*_iso_(H) = 1.2*U*_eq_(O).

### Crystal data

**Table:**

C_21_H_26_N_2_O_4_	*Z* = 2
*M**_r_* = 370.44	*F*(000) = 396
Triclinic, *P*1	*D*_x_ = 1.238 Mg m^−^^3^
Hall symbol: -P 1	Mo *K*α radiation, λ = 0.71073 Å
*a* = 9.140 (3) Å	Cell parameters from 1361 reflections
*b* = 11.451 (4) Å	θ = 2.4–23.9°
*c* = 13.013 (5) Å	µ = 0.09 mm^−^^1^
α = 113.845 (5)°	*T* = 273 K
β = 109.628 (6)°	Block, yellow
γ = 108.812 (5)°	0.12 × 0.11 × 0.09 mm
*V* = 993.5 (6) Å^3^	

### Data collection

**Table:**

Bruker APEXII CCD area-detector diffractometer	3451 independent reflections
Radiation source: fine-focus sealed tube	2325 reflections with *I* > 2σ(*I*)
graphite	*R*_int_ = 0.030
φ and ω scans	θ_max_ = 25.0°, θ_min_ = 2.0°
Absorption correction: multi-scan (*SADABS*; Sheldrick, 1996)	*h* = −10→10
*T*_min_ = 0.990, *T*_max_ = 0.992	*k* = −13→11
4894 measured reflections	*l* = −15→13

### Refinement

**Table:**

Refinement on *F*^2^	Primary atom site location: structure-invariant direct methods
Least-squares matrix: full	Secondary atom site location: difference Fourier map
*R*[*F*^2^ > 2σ(*F*^2^)] = 0.066	Hydrogen site location: inferred from neighbouring sites
*wR*(*F*^2^) = 0.193	H-atom parameters constrained
*S* = 1.00	*w* = 1/[σ^2^(*F*_o_^2^) + (0.1202*P*)^2^] where *P* = (*F*_o_^2^ + 2*F*_c_^2^)/3
3451 reflections	(Δ/σ)_max_ < 0.001
249 parameters	Δρ_max_ = 0.26 e Å^−^^3^
0 restraints	Δρ_min_ = −0.26 e Å^−^^3^

### Special details

**Table:**

Geometry. All e.s.d.'s (except the e.s.d. in the dihedral angle between two l.s. planes) are estimated using the full covariance matrix. The cell e.s.d.'s are taken into account individually in the estimation of e.s.d.'s in distances, angles and torsion angles; correlations between e.s.d.'s in cell parameters are only used when they are defined by crystal symmetry. An approximate (isotropic) treatment of cell e.s.d.'s is used for estimating e.s.d.'s involving l.s. planes.
Refinement. Refinement of *F*^2^ against ALL reflections. The weighted *R*-factor *wR* and goodness of fit *S* are based on *F*^2^, conventional *R*-factors *R* are based on *F*, with *F* set to zero for negative *F*^2^. The threshold expression of *F*^2^ > σ(*F*^2^) is used only for calculating *R*-factors(gt) *etc*. and is not relevant to the choice of reflections for refinement. *R*-factors based on *F*^2^ are statistically about twice as large as those based on *F*, and *R*- factors based on ALL data will be even larger.

### Fractional atomic coordinates and isotropic or equivalent isotropic displacement parameters (Å^2^)

**Table:**

	*x*	*y*	*z*	*U*_iso_*/*U*_eq_	
O1	0.2732 (2)	0.85334 (19)	0.31097 (15)	0.0615 (5)	
H1	0.2881	0.8851	0.3848	0.092*	
O2	0.1699 (2)	0.75251 (19)	0.06238 (15)	0.0625 (5)	
O3	0.1229 (2)	0.44342 (19)	0.38472 (16)	0.0658 (5)	
H3	0.1614	0.5336	0.4307	0.099*	
O4	0.0549 (2)	0.16836 (19)	0.25933 (17)	0.0666 (5)	
N1	0.1898 (3)	0.8527 (2)	0.4813 (2)	0.0553 (5)	
N2	0.3270 (3)	0.7183 (2)	0.60118 (19)	0.0571 (5)	
C1	−0.0337 (3)	0.6987 (3)	0.2479 (2)	0.0521 (6)	
C2	0.0950 (3)	0.7499 (2)	0.2166 (2)	0.0477 (6)	
C3	0.0365 (3)	0.6931 (3)	0.0830 (2)	0.0514 (6)	
C4	−0.1454 (3)	0.5848 (3)	−0.0147 (3)	0.0621 (7)	
H4	−0.1842	0.5446	−0.1036	0.075*	
C5	−0.2713 (4)	0.5348 (3)	0.0171 (3)	0.0717 (8)	
H5	−0.3937	0.4618	−0.0504	0.086*	
C6	−0.2174 (3)	0.5916 (3)	0.1466 (3)	0.0673 (7)	
H6	−0.3033	0.5588	0.1674	0.081*	
C7	0.0251 (4)	0.7553 (3)	0.3859 (3)	0.0554 (6)	
H7	−0.0619	0.7182	0.4045	0.067*	
C8	0.1151 (4)	0.6978 (3)	−0.0732 (2)	0.0642 (7)	
H8A	0.0269	0.7214	−0.1103	0.077*	
H8B	0.0583	0.5892	−0.1270	0.077*	
C9	0.2824 (4)	0.7724 (3)	−0.0729 (3)	0.0811 (9)	
H9A	0.3376	0.8797	−0.0192	0.122*	
H9B	0.2492	0.7382	−0.1626	0.122*	
H9C	0.3681	0.7475	−0.0369	0.122*	
C10	0.3713 (3)	0.5196 (3)	0.5889 (2)	0.0523 (6)	
C11	0.2296 (3)	0.4113 (3)	0.4549 (2)	0.0509 (6)	
C12	0.1958 (3)	0.2656 (3)	0.3898 (2)	0.0543 (6)	
C13	0.3036 (4)	0.2294 (3)	0.4590 (3)	0.0641 (7)	
H13	0.2816	0.1324	0.4161	0.077*	
C14	0.4442 (4)	0.3374 (3)	0.5920 (3)	0.0722 (8)	
H14	0.5158	0.3124	0.6379	0.087*	
C15	0.4778 (4)	0.4803 (3)	0.6557 (3)	0.0687 (7)	
H15	0.5728	0.5520	0.7446	0.082*	
C16	0.4133 (3)	0.6729 (3)	0.6564 (2)	0.0563 (6)	
H16	0.5092	0.7418	0.7454	0.068*	
C17	0.0096 (4)	0.0166 (3)	0.1890 (3)	0.0718 (8)	
H17A	−0.0139	−0.0273	0.2360	0.086*	
H17B	0.1100	0.0128	0.1810	0.086*	
C18	−0.1577 (5)	−0.0678 (3)	0.0545 (3)	0.0911 (10)	
H18A	−0.2550	−0.0608	0.0637	0.137*	
H18B	−0.1943	−0.1717	0.0050	0.137*	
H18C	−0.1316	−0.0253	0.0079	0.137*	
C19	0.3894 (4)	0.8780 (3)	0.6768 (2)	0.0609 (7)	
H19A	0.4958	0.9345	0.6779	0.073*	
H19B	0.4262	0.9145	0.7681	0.073*	
C20	0.2425 (4)	0.9054 (3)	0.6181 (2)	0.0606 (7)	
H20	0.1354	0.8498	0.6181	0.073*	
C21	0.3133 (5)	1.0728 (3)	0.6992 (3)	0.0823 (9)	
H21A	0.4190	1.1274	0.7001	0.123*	
H21B	0.3464	1.1076	0.7889	0.123*	
H21C	0.2192	1.0891	0.6599	0.123*	

### Atomic displacement parameters (Å^2^)

**Table:**

	*U*^11^	*U*^22^	*U*^33^	*U*^12^	*U*^13^	*U*^23^
O1	0.0559 (10)	0.0630 (11)	0.0471 (9)	0.0206 (9)	0.0246 (9)	0.0290 (9)
O2	0.0652 (11)	0.0655 (11)	0.0460 (9)	0.0286 (9)	0.0262 (8)	0.0316 (9)
O3	0.0776 (12)	0.0552 (11)	0.0516 (10)	0.0405 (10)	0.0184 (9)	0.0305 (9)
O4	0.0835 (12)	0.0521 (10)	0.0561 (11)	0.0391 (10)	0.0296 (10)	0.0292 (9)
N1	0.0655 (13)	0.0568 (12)	0.0575 (12)	0.0359 (11)	0.0376 (11)	0.0371 (11)
N2	0.0645 (12)	0.0497 (12)	0.0481 (12)	0.0273 (10)	0.0254 (10)	0.0279 (10)
C1	0.0525 (13)	0.0543 (14)	0.0617 (15)	0.0350 (12)	0.0305 (12)	0.0374 (13)
C2	0.0481 (13)	0.0457 (13)	0.0480 (13)	0.0267 (11)	0.0205 (11)	0.0290 (11)
C3	0.0581 (14)	0.0496 (14)	0.0507 (14)	0.0336 (12)	0.0263 (12)	0.0305 (12)
C4	0.0612 (16)	0.0656 (17)	0.0531 (15)	0.0383 (14)	0.0199 (13)	0.0346 (13)
C5	0.0499 (15)	0.0744 (19)	0.0703 (19)	0.0333 (14)	0.0175 (14)	0.0382 (16)
C6	0.0514 (15)	0.0750 (18)	0.0800 (19)	0.0363 (14)	0.0324 (15)	0.0483 (16)
C7	0.0610 (15)	0.0584 (15)	0.0669 (16)	0.0367 (13)	0.0408 (14)	0.0419 (14)
C8	0.0781 (17)	0.0652 (16)	0.0425 (13)	0.0379 (14)	0.0274 (13)	0.0302 (13)
C9	0.095 (2)	0.085 (2)	0.0584 (17)	0.0396 (18)	0.0421 (16)	0.0423 (16)
C10	0.0522 (13)	0.0598 (15)	0.0498 (13)	0.0284 (12)	0.0264 (12)	0.0370 (12)
C11	0.0558 (13)	0.0574 (15)	0.0537 (14)	0.0337 (12)	0.0300 (12)	0.0394 (12)
C12	0.0609 (15)	0.0575 (15)	0.0559 (15)	0.0335 (13)	0.0342 (13)	0.0372 (13)
C13	0.0728 (17)	0.0681 (17)	0.0798 (19)	0.0450 (15)	0.0460 (16)	0.0538 (16)
C14	0.0699 (17)	0.083 (2)	0.083 (2)	0.0471 (16)	0.0342 (16)	0.0630 (18)
C15	0.0634 (16)	0.0759 (19)	0.0646 (16)	0.0350 (15)	0.0245 (14)	0.0484 (15)
C16	0.0538 (14)	0.0562 (15)	0.0439 (13)	0.0209 (12)	0.0201 (12)	0.0291 (12)
C17	0.0860 (19)	0.0567 (17)	0.079 (2)	0.0429 (16)	0.0472 (17)	0.0374 (15)
C18	0.106 (2)	0.0601 (18)	0.080 (2)	0.0436 (18)	0.041 (2)	0.0271 (17)
C19	0.0686 (15)	0.0513 (15)	0.0475 (14)	0.0251 (13)	0.0255 (12)	0.0271 (12)
C20	0.0739 (17)	0.0623 (16)	0.0578 (15)	0.0367 (14)	0.0445 (14)	0.0359 (13)
C21	0.114 (2)	0.0701 (19)	0.081 (2)	0.0571 (19)	0.063 (2)	0.0424 (17)

### Geometric parameters (Å, °)

**Table:**

O1—C2	1.341 (3)	C9—H9B	0.9600
O1—H1	0.8200	C9—H9C	0.9600
O2—C3	1.361 (3)	C10—C15	1.391 (3)
O2—C8	1.437 (3)	C10—C11	1.397 (3)
O3—C11	1.351 (3)	C10—C16	1.447 (3)
O3—H3	0.8200	C11—C12	1.394 (3)
O4—C12	1.364 (3)	C12—C13	1.387 (4)
O4—C17	1.423 (3)	C13—C14	1.388 (4)
N1—C7	1.262 (3)	C13—H13	0.9300
N1—C20	1.461 (3)	C14—C15	1.366 (4)
N2—C16	1.263 (3)	C14—H14	0.9300
N2—C19	1.461 (3)	C15—H15	0.9300
C1—C6	1.393 (3)	C16—H16	0.9300
C1—C2	1.401 (3)	C17—C18	1.489 (4)
C1—C7	1.456 (4)	C17—H17A	0.9700
C2—C3	1.404 (3)	C17—H17B	0.9700
C3—C4	1.376 (3)	C18—H18A	0.9600
C4—C5	1.382 (4)	C18—H18B	0.9600
C4—H4	0.9300	C18—H18C	0.9600
C5—C6	1.362 (4)	C19—C20	1.503 (4)
C5—H5	0.9300	C19—H19A	0.9700
C6—H6	0.9300	C19—H19B	0.9700
C7—H7	0.9300	C20—C21	1.521 (4)
C8—C9	1.490 (4)	C20—H20	0.9800
C8—H8A	0.9700	C21—H21A	0.9600
C8—H8B	0.9700	C21—H21B	0.9600
C9—H9A	0.9600	C21—H21C	0.9600
			
C2—O1—H1	109.5	O4—C12—C13	124.9 (2)
C3—O2—C8	116.92 (19)	O4—C12—C11	115.6 (2)
C11—O3—H3	109.5	C13—C12—C11	119.5 (2)
C12—O4—C17	118.2 (2)	C12—C13—C14	120.2 (3)
C7—N1—C20	120.5 (2)	C12—C13—H13	119.9
C16—N2—C19	118.7 (2)	C14—C13—H13	119.9
C6—C1—C2	119.9 (2)	C15—C14—C13	120.3 (2)
C6—C1—C7	120.0 (2)	C15—C14—H14	119.9
C2—C1—C7	120.1 (2)	C13—C14—H14	119.9
O1—C2—C1	122.0 (2)	C14—C15—C10	120.7 (3)
O1—C2—C3	118.7 (2)	C14—C15—H15	119.6
C1—C2—C3	119.4 (2)	C10—C15—H15	119.6
O2—C3—C4	125.7 (2)	N2—C16—C10	123.6 (2)
O2—C3—C2	115.3 (2)	N2—C16—H16	118.2
C4—C3—C2	119.1 (2)	C10—C16—H16	118.2
C3—C4—C5	121.1 (2)	O4—C17—C18	107.3 (2)
C3—C4—H4	119.4	O4—C17—H17A	110.3
C5—C4—H4	119.4	C18—C17—H17A	110.3
C6—C5—C4	120.5 (2)	O4—C17—H17B	110.3
C6—C5—H5	119.8	C18—C17—H17B	110.3
C4—C5—H5	119.8	H17A—C17—H17B	108.5
C5—C6—C1	120.0 (3)	C17—C18—H18A	109.5
C5—C6—H6	120.0	C17—C18—H18B	109.5
C1—C6—H6	120.0	H18A—C18—H18B	109.5
N1—C7—C1	122.4 (2)	C17—C18—H18C	109.5
N1—C7—H7	118.8	H18A—C18—H18C	109.5
C1—C7—H7	118.8	H18B—C18—H18C	109.5
O2—C8—C9	107.9 (2)	N2—C19—C20	112.0 (2)
O2—C8—H8A	110.1	N2—C19—H19A	109.2
C9—C8—H8A	110.1	C20—C19—H19A	109.2
O2—C8—H8B	110.1	N2—C19—H19B	109.2
C9—C8—H8B	110.1	C20—C19—H19B	109.2
H8A—C8—H8B	108.4	H19A—C19—H19B	107.9
C8—C9—H9A	109.5	N1—C20—C19	109.1 (2)
C8—C9—H9B	109.5	N1—C20—C21	109.2 (2)
H9A—C9—H9B	109.5	C19—C20—C21	110.2 (2)
C8—C9—H9C	109.5	N1—C20—H20	109.5
H9A—C9—H9C	109.5	C19—C20—H20	109.5
H9B—C9—H9C	109.5	C21—C20—H20	109.5
C15—C10—C11	119.3 (2)	C20—C21—H21A	109.5
C15—C10—C16	119.9 (2)	C20—C21—H21B	109.5
C11—C10—C16	120.8 (2)	H21A—C21—H21B	109.5
O3—C11—C12	118.1 (2)	C20—C21—H21C	109.5
O3—C11—C10	121.8 (2)	H21A—C21—H21C	109.5
C12—C11—C10	120.0 (2)	H21B—C21—H21C	109.5

### Hydrogen-bond geometry (Å, °)

**Table:**

*D*—H···*A*	*D*—H	H···*A*	*D*···*A*	*D*—H···*A*
O3—H3···N2	0.82	1.89	2.614 (3)	147
O1—H1···N1	0.82	1.85	2.576 (2)	146
